# Using GIS to Estimate Population at Risk Because of Residence Proximity to Asbestos Processing Facilities in Colombia

**DOI:** 10.3390/ijerph182413297

**Published:** 2021-12-17

**Authors:** Benjamin Lysaniuk, María Fernanda Cely-García, Margarita Giraldo, Joan M. Larrahondo, Laura Marcela Serrano-Calderón, Juan Carlos Guerrero-Bernal, Leonardo Briceno-Ayala, Esteban Cruz Rodriguez, Juan Pablo Ramos-Bonilla

**Affiliations:** 1 IRD (MàD by CNRS)—UMR Prodig, 93222 Aubervilliers, France; Benjamin.Lysaniuk@cnrs.fr; 2 Departamento de Ingeniería Civil y Ambiental, Universidad de Los Andes, Bogotá 111711, Colombia; mf.cely46@uniandes.edu.co (M.F.C.-G.); mm.giraldo337@uniandes.edu.co (M.G.); 3 Departamento de Ingeniería Civil, Facultad de Ingeniería, Pontificia Universidad Javeriana, Bogotá 110231, Colombia; jlarrahondo@javeriana.edu.co (J.M.L.); lauramserranoc@gmail.com (L.M.S.-C.); 4 Facultad de Estudios Internacionales, Políticos y Urbanos (FEIPU), Universidad del Rosario, Bogotá 111711, Colombia; juan.guerrero@urosario.edu.co (J.C.G.-B.); esteban.cruz@urosario.edu.co (E.C.R.); 5 Escuela de Medicina y Ciencias de la Salud, Universidad del Rosario, Bogotá 111711, Colombia; leonardo.briceno@urosario.edu.co

**Keywords:** asbestos, environmental exposure, population, geographic information system, Colombia

## Abstract

The recent enactment of the law banning asbestos in Colombia raises a significant number of challenges. The largest factories that have historically processed asbestos include five asbestos-cement facilities located in the cities of Sibaté (Cundinamarca), Cali (Valle del Cauca), and Barranquilla (Atlántico), and Manizales (Caldas), which has two, as well as a friction products facility in Bogotá D.C. An asbestos chrysotile mine has also operated in Colombia since 1980 in Campamento (Antioquia). In the framework of developing the National Asbestos Profile for Colombia, in this study, we estimated the population residing in the vicinity of asbestos processing plants or the mine and, therefore, potentially at risk of disease. Using a geographic information system, demographic data obtained from the last two general population censuses were processed to determine the number of people living within the concentric circles surrounding the asbestos facilities and the mine. In previous studies conducted in different countries of the world, an increased risk of asbestos-related diseases has been reported for people living at different distance bands from asbestos processing facilities. Based on these studies, circles of 500, 1000, 2000, 5000, and 10,000 m radii, centered on the asbestos processing facilities and the mine that operated in Colombia, were combined with the census data to estimate the number of people living within these radii. Large numbers of people were identified. It is estimated that in 2005, at the country level, 10,489 people lived within 500 m of an asbestos processing facility or mine. In 2018, and within a distance of 10,000 m, the number of people was 6,724,677. This information can aid public health surveillance strategies.

## 1. Introduction

Asbestos minerals are classified into two groups, namely amphiboles and serpentines [1]. Amphiboles include five asbestos minerals (amosite, actinolite, anthophyllite, tremolite, and crocidolite), and serpentines include chrysotile, which is currently the type of asbestos most extensively used worldwide [1,2,3,4]. Because of their physical and chemical characteristics, asbestos minerals have been used in many products and in several industrial sectors [5,6]. All types of asbestos are carcinogenic to humans (i.e., type I carcinogens), and exposure to this material causes diseases such as mesothelioma, lung cancer, laryngeal cancer, ovarian cancer, and asbestosis [7,8]. However, it is difficult to calculate the burden of disease related to the population attributable fraction of these multifactorial diseases because of limitations in the asbestos-related environmental and health data [9], especially in low- and middle-income countries. Previous studies have shown that the risk for lung cancer from asbestos exposure is variable according to the type of exposure (environmental, domestic, or occupational) [10], level of exposure, the relative toxicity of asbestos type [11], awareness of the health and socio-economic impact of asbestos use and bans [12], and the level of release of fibers from materials, among other variables [13]. Unfortunately, this type of information is not always available in low- and middle-income countries, as is the case for Colombia. The highest levels of asbestos exposure and the burden of asbestos-related diseases occur primarily in occupationally exposed workers, but it is important to acknowledge that the role of non-occupational exposure cannot be neglected [1,14]. 

Despite the negative effects of asbestos on human health, products containing asbestos are still sold in many countries. As of 2021, only 67 countries, a group that Colombia joined recently, have banned this mineral [15]. In July 2019, Law 1968 was enacted in Colombia, banning the use of all asbestos types [16]. The law establishes that starting 1 January 2021, all activities related to asbestos are prohibited, including its mining, production, selling, importation, distribution, and exportation. This is undoubtedly one of the most important achievements in recent years in the field of public health in Colombia, as it helps to curb the growth of the asbestos problem. However, it is important to understand that Law 1968/19 by itself is not the solution to the asbestos problem, and among other issues, the asbestos products that have been distributed throughout the country for 78 years still represent a major health threat and an important technical and economic challenge. In fact, Law 1968/19 stipulates that the Colombian government has five years from the enactment of the law to formulate a strategy to address this problem. One of the conditions making it more difficult for strategy designs to face the challenges imposed by the use of asbestos in Colombia is the lack of information regarding the magnitude and characteristics of the problem. 

The World Health Organization (WHO) and the International Labour Organization (ILO) have recommended that countries implement National Programs for the Elimination of Asbestos-Related Diseases (NPEAD), a strategy designed to address the asbestos problem at the country level [17]. The preparation of NPEAD begins with the development of the National Asbestos Profile (NAP), which includes 18 sections that describe the country’s legislative context, the production and trade of mineral asbestos and asbestos-containing products, the industries with the highest risk of exposure and high-risk workers, the incidence and prevalence of asbestos-related diseases, the occupational exposure limits of the country, the studies conducted on the topic in the country, and estimated economic losses due to asbestos-related diseases [17]. This study is part of an effort to develop the NAP of Colombia, which is currently in development by a multidisciplinary group of engineers, geologists, physicians, geographers, and political scientists from three Colombian Universities and a French public research institution.

Abundant scientific literature shows that populations that are settled nearby industrial asbestos processing facilities and mines are at excessive risk of developing asbestos-related diseases. The cases of Casale Monferrato (Italy) [18,19], Amagasaki (Japan) [20,21], Wittenoom (Australia) [22], Libby (USA) [23]—among other examples—are particularly well documented. 

Six major facilities and an asbestos mine, shown in Table 1, have operated in Colombia and were the focus of the current study.

The asbestos processing facilities include five asbestos cement facilities that manufactured construction products (e.g., corrugated asbestos cement sheets, pipes for water supply and sanitation systems, and water storage tanks), and one friction products plant that manufactured products for the automotive sector (e.g., brake pads, brake linings, brake blocks, and clutch discs). There is also a chrysotile asbestos mine located in Campamento (Department of Antioquia). 

Using a geographic information system (GIS) to process census records for Colombia from different years, in this study, we estimated the number of people from the general population living in distance bands from asbestos processing facilities and the asbestos mine at which an elevated risk of asbestos-related diseases (ARD) has been reported in the scientific literature. 

## 2. Materials and Methods

To determine the distances around asbestos facilities or asbestos mines at which an elevated risk of asbestos-related diseases has been observed, a literature review was performed to identify studies that had conducted this type of analysis. The literature review was conducted in PubMed at the end of 2020, using the search words “asbestos”, “distance”, “environmental exposure” and “plant OR factory”, with no limits on the publication year. 

Using a GIS, the distances identified in the literature review were cross-checked with demographic information obtained from the last two general censuses of the Colombian population (i.e., 2005 and 2018) developed by the National Administrative Department of Statistics (Departamento Administrativo Nacional de Estadística—DANE) [24]. These censuses are the only ones available online. To estimate the population living within these distances, the National Geostatistical Framework of Colombia (Marco Geoestadistico Nacional—MGN) [25] was used. This database is organized following the political and administrative divisions of Colombia and includes demographic information that is very specific from a geographical perspective. 

All administrative subdivisions of Colombia are characterized by a unique identifier appearing both in the MGN and in the DANE databases. Thus, it was possible to integrate the relevant census data into each polygon representing the administrative units of interest for 2005 and 2018. Demographic data in Colombia is divided between urban and rural areas. Considering the radii used, it was, therefore, necessary to integrate the urban and rural areas to minimize the risk of population loss in the estimates. To do this, the smallest administrative level for each of the censuses was used (i.e., sections and/or sectors) according to the following classification:

- For the year 2005: population counts by five-year age groups for urban sections and rural sectors.
- For the year 2018: population counts by five-year age groups for urban and rural sections.

The urban sections for the 2005 census correspond to the second smallest level of data in urban areas. The city blocks (i.e., manzanas) are the most precise areas, but because of the large amount of data that the DANE servers had to process at the city block level, an error message was always generated when conducting the analysis at this level. Furthermore, since large amounts of data were missing from rural sections for 2005, the next size level for rural areas (i.e., rural sectors) was used. The urban and rural sections of the 2018 census correspond to the most precise level of data in both types of areas for that year. The procedure for obtaining the data used in the analysis is detailed in the Supplementary Material, Figure S1.

As explained before, the analysis was conducted in the regions where asbestos facilities or the mine are located, including the departments of Antioquia (Las Brisas Mine in the municipality of Campamento), Atlántico (Eternit in the municipality of Barranquilla), Bogotá D.C. (Incolbest in the Capital District), Caldas (Etex and Toptec in the municipality of Manizales), Cundinamarca (Eternit in the municipality of Sibaté), and Valle del Cauca (Eternit in the municipality of Yumbo). Data from the Departments of Magdalena and Tolima were also integrated into the analysis of the populations around Eternit, Barranquilla and Etex, Manizales respectively, since small areas of these departments were located within some radii of interest. 

After compiling all the data, it was processed with MGN shapefiles using Arcgis 10.8 (©Esri). The procedure used included the following steps (the complete procedure is detailed in the Supplementary Material, Figure S2):

- Location of the asbestos facilities and the mine using satellite images integrated into a GIS via a web server and the creation of a point for each site.
- Creation of buffer zones using the distances identified in the literature review.
- Calculation of the individual area of the MGN polygons (area A).
- Use of the “intersection” tool under Arcgis between each buffer zone and the polygons of the urban sections (2005 and 2018), rural sectors (2005), and rural sections (2018).
- Calculation of the area of the polygons resulting from the intersection between the buffers and the MGN layers (area B).
- Calculation of the ratio between area B and area A. This ratio was applied to the census populations in cases in which polygons of the MGN were not fully covered by a buffer.
- Integration of the census data by table joining the DANE data and the “buffer/MGN polygons” intersection layers.
- Exportation of the attribute tables in text format, followed by post-processing (i.e., the application of the ratio between area B to area A and the corresponding population), and estimation of the number of people residing there, categorized in five-year age groups, within the concentric radii surrounding the asbestos processing facilities and the mine. This procedure was done for both years 2005 and 2018. When fractional numbers were obtained for the population estimates, the value was rounded to the nearest integer, which may result in some slight differences in the calculations of row and column sums in the tables presented in the results.

The Integrated Information System of Social Protection (SISPRO), the official database of morbidity and mortality managed by the Colombian Ministry of Health and Social Protection, was also consulted to determine if it was possible to find cases of mesothelioma, a sentinel disease of asbestos exposure, in the six municipalities where the analysis was conducted. SISPRO was accessed on 5 October 2021. Three Excel pivot tables were built, filtering by the “main diagnosis” of pleural, peritoneal, and pericardial mesothelioma (i.e., CIE10 450, 451, and 452, respectively). Searches were made at the municipality level for Campamento (Antioquia), Barranquilla (Atlántico), Bogotá (DC), Manizales (Caldas), Sibaté (Cundinamarca), and Yumbo (Valle del Cauca). Gender, date of healthcare provided, and age at the moment of the healthcare provided (i.e., by 5-year age groups) were also included in the search. The numerical variable used was “number of people served”, which shows the number of persons served by the healthcare system at a particular moment and locations with specific diseases within a specific window of time, clarifying that because of how the database works, more than one healthcare visit may be reported for the same person. Since the information in SISPRO can only be consulted at the municipality level, and the exact current and past locations of residence of the cases are not available in the database, it was not possible to conduct a more detailed geographic analysis of the exact location of the mesothelioma cases identified by SISPRO.

## 3. Results

In the literature review, seven studies that analyzed the risk of developing malignant mesothelioma at different distances from an industrial emission source point, generally an asbestos-cement factory, were identified [19,21,26,27,28,29,30]. The studies are presented in Table 2. The distances considered in these articles refer to radii relative to the circles centered on the point of emission (e.g., an asbestos-cement facility). For mining activities, several articles were identified for Libby (USA) [31,32], Wittenoom (Australia) [33,34], and South Africa [35,36]. Unfortunately, an analysis of the risk of asbestos-related diseases in relation to the distance to the mine was not conducted, and it was not possible to identify this type of analysis in regions where asbestos mines have operated. In the case of Libby, Noonan [37] found that local residents suffered from elevated mesothelioma incidence rates between 1.5 and 11 km from the mine. However, the study recognized that it was not possible to determine if the elevated rates were the result of atmospheric dispersion from the mine or from asbestos residues deposited in residential areas, as it was previously discussed for the same site [32]. Thus, for the current study, to estimate the number of people that live in the vicinity of the asbestos mine, the same distance bands around asbestos processing facilities for which the literature indicates an increased risk of malignant mesothelioma were applied.

In summary, for the current analysis, the distances used were radii of 500, 1000, 2000, 5000, and 10,000 m, as shown in Table 2, which also includes the health risk estimates reported in the studies. In Table 2, it is possible to observe great variations in the risk estimates. When comparing the magnitude of such risk estimates, there are no clear tendencies among the studies. This is expected since the study designs, local contexts, and asbestos facility characteristics differ, conferring unique features for each study and, consequently, unique findings. 

**Figure 1:**
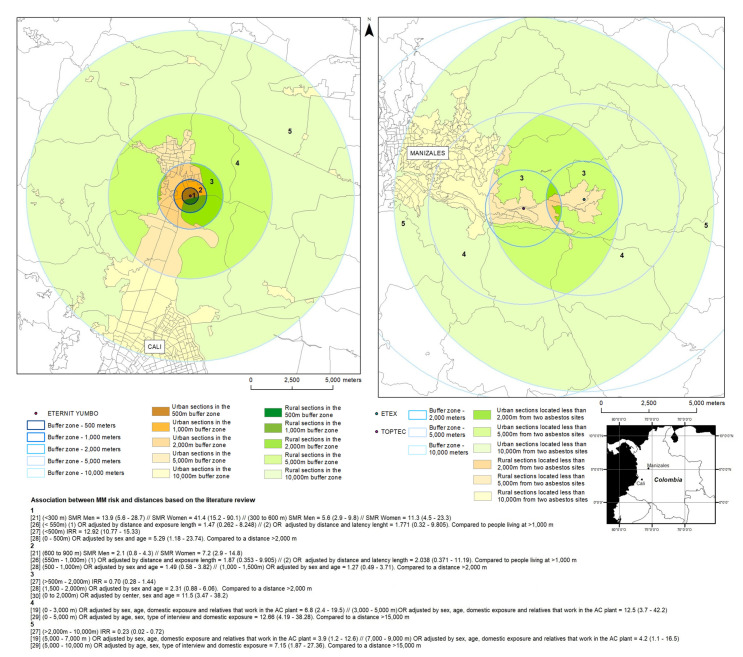
Illustration of the intersection between the buffer zones, based on the relationship between “distance bands and MM risk” extracted from the literature, and the polygons of the MGN (left—Eternit, Yumbo, 2018), highlighting the sectors potentially impacted by atmospheric releases from two sites (right—Etex and Toptec—Manizales, Caldas, 2018).

Cross-referencing the demographic data from the DANE with that of the MGN was done to estimate the populations living within distance bands around the six asbestos processing facilities and the mine, and the results are presented in Table 3 (with 2005 census data) and Table 4 (with 2018 census data). Figure 1 presents two maps of the buffer zones created, and the associated mesothelioma risk reported for each distance band, in the municipality of Yumbo, surrounding an asbestos cement plant (Eternit Pacífico), and in Manizales, surrounding two asbestos cement plants (Etex and Toptec).

For both 2005 and 2018, the population potentially at risk was—like the Colombian population—relatively young. In 2005, 27.5% of the people living within a radius of 10,000 m around the sites were between 0 and 14 years old, which is similar—for the same year—to the percentage of this age group for the entire Colombian population (28.94%) [38]. In 2018, this proportion of very young people living within a 10,000 m radius fell to 20%, which is slightly lower compared to the percentage of people in this age group for the entire Colombian population (23.07%) [38].

It is also important to highlight that six of the seven locations analyzed were either within an urban center or in close proximity to urban centers, with the exception of the mine, which is located in a remote rural area. This explains the large number of people identified, which obviously increased as the radii increased. With the exception of the mine in Campamento, with a total of 2724 inhabitants in 2005 and 8472 inhabitants in 2018 living within a 10 km radius of the mine (Table 3 and Table 4), all the other sites analyzed resulted in population numbers in the order of hundreds of thousands or even millions of people. For the 2005 census, the number of people living in a 10 km radius of an asbestos processing facility ranged from 282,479 around Etex (Manizales) to 3,346,920 around Incolbest (Bogotá), and for the 2018 census, the range was from 312,615 around Etex (Manizales) to 3709,324 around Incolbest (Bogotá) (Table 3 and Table 4).

Another important aspect of this analysis is that there were two regions that had two asbestos processing plants; one region was the city of Manizales (Caldas), with the asbestos cement plants of Etex and Toptec, and the other was the Department of Cundinamarca where the Capital District of Bogotá is located, with the Incolbest friction products plant in Bogotá and the asbestos cement plant of Eternit in Sibaté. Thus, the number of people living within the area of influence of two plants was identified. In conducting this analysis, it was determined that 282,175 inhabitants lived less than 10,000 m from both the Etex and Toptec plants in Manizales in 2005 (312,554 people in 2018), and 38,728 people lived less than 10,000 m from both the Incolbest (Bogotá) and Eternit (Sibaté) plants in 2005 (51,831 people in 2018) (Table 5 and Table 6).

On the national scale and considering all the sites of interest for this study, the number of people living within a 10 km radius around these sites is in the millions. Highlighting that no double counting of people was done in Manizales or Bogotá/Sibaté, it is estimated that in 2005, 5,926,382 people lived within 10 km of the seven sites analyzed, and for 2018, this number reached 6,724,677 people. To put the previous figures into context, the population of Colombia was 41,468,384 in 2005 and 48,258,494 in 2018 [24]. Furthermore, the total number of people living within a radius of 1000 m from all seven sites was 53,904 in 2005, and 59,459 in 2018 (Table 7).

It is important to recognize that in the demographic data in the DANE database, there were some polygons with no information, for both 2005 and 2018 (Supplementary Material, Figure S3a,b). It should be noted that the general population census seems to have gained in quality between 2005 and 2018 since the number of polygons without data was drastically reduced. Moreover, it is possible that the lack of data in some polygons has resulted in an underestimation of the populations at risk, based on several official communications with the DANE.

Table 8 shows the number of cases attended to by a healthcare provider at the municipality level between 1 January 2015 and 30 June 2021. A total of 572 registries of health services that attended to mesothelioma cases (i.e., pleural, peritoneal, and pericardial) were identified for the period analyzed in the six municipalities included in the current study (i.e., Campamento, Barranquilla, Bogotá, Manizales, Sibaté, and Yumbo). The age at the moment of the provided healthcare (i.e., by 5-year age groups) was also included in the search (i.e., not shown in Table 8). Because of how the database works, more than one healthcare visit for the same person could be reported for the period consulted. Thus, as is shown in Table 8, for pleural mesothelioma, the total number of people attended to was 350, but the number of visits was 356. The information presented in Table 8 is focused on mesotheliomas of the pleura (C450), peritoneum (C451), and pericardium (C452), as these are sentinel diseases of asbestos exposure. However, these are not necessarily incident cases, and therefore, risk estimates, such as age-adjusted incident rates, cannot be derived from these figures. Although the cases reported in Table 8 were identified using the municipality of residence of the cases as one of the filters, (that is, instead of the location of the health center where the diagnosis or treatment took place), it is possible, as it was observed in a previous study in Sibaté [39], that the residence of the patient is not properly introduced in the SISPRO database. This could explain why in Campamento, a location where an asbestos chrysotile mine operated for more than four decades, no mesothelioma cases were observed, although this could also be explained by the latency and rarity of the disease. Additionally, in the figures consulted, there were mesothelioma cases at unusually young ages (not shown in the table), including cases in the age group of 0–4 years old. Moreover, in the municipality of Yumbo, no pleural, peritoneal, and pericardial mesothelioma cases were reported in women for the time window analyzed, which could be an artifact of the database. Finally, specific information for each case, such as home address and occupation are not reported in SISPRO, which impedes conducting a more sophisticated epidemiological analysis. Thus, the numbers reported in Table 8 should be used with caution.

## 4. Discussion

The operation of seven facilities that process asbestos in Colombia, the first one beginning in 1942, has left a negative legacy of asbestos-containing products in the country, as well as people occupationally and environmentally exposed to asbestos. Furthermore, a malignant pleural mesothelioma cluster involving, so far, mostly members of the general population was found in Sibaté, where the first asbestos cement facility of the country was built [39]. The potential presence of an excess number of asbestos-related diseases in other regions in Colombia where asbestos processing facilities have operated has not yet been assessed. In the current study, the number of people living in the vicinity of asbestos processing plants or the mine ranges from approximately 10,000 inhabitants within the 500 m radii to about 6 million people within the 10 km radii, the latter representing about 14% of the country’s total population. This clearly indicates the importance of establishing surveillance programs for both asbestos-related diseases and asbestos exposure sources in regions where asbestos processing plants and the mine have operated, something that has become more relevant considering the large number of people living nearby these facilities.

This analysis, as rigorous as it was, should be read only as an estimation exercise, considering that it was based on distances at which an increased risk of ARD has been observed in other locations of the world, but no asbestos exposure campaigns have been conducted in Colombia in the surroundings of asbestos facilities or the mine, similarly to what happens in most low- and middle-income countries. Only sampling campaigns could prove if the real exposure to asbestos fibers in these areas occurred, as it has been exemplified by several cases, including serpentine quarries in Italy [40], an asbestos textile factory in Indonesia [41], and an asbestos cement sheet manufacturing plant in India [42]. However, since asbestos has already been banned in Colombia, the opportunity to measure asbestos concentrations in the air surrounding asbestos processing facilities and the mine is no longer available. This shows the importance of measuring asbestos fiber concentrations, implementing air quality models, and implementing asbestos-related disease surveillance programs in regions of the world where asbestos processing facilities or asbestos mines are still operating.

Another point to mention is that the population residing around the Campamento mine has increased much more in proportion than those around the other sites during the 2005–2018 period (Table 3 and Table 4), indicating that the 2005 census may have missed inhabitants of the rural area (Supplementary Material, Figure S3a). This is important because there was a significant increase in asbestos production from the mine between the years 2005 and 2018. In 2005 and the five years preceding the 2005 census, the asbestos production of the mine ranged between 4246 and 6539 tons of asbestos per year [43,44]. In 2018 and the five years preceding the 2018 census, the production of the mine increased to a range between 3550 and 13,239 tons of asbestos per year, an increase that coincides with a new mine owner taking over in 2013 [45]. Thus, what seems to be a significant increase in the population at risk in the region of the Campamento mine is consistent with a significant increase in chrysotile fiber production.

The present study has the following limitations. First, in some cases, the polygons of the MGN were not entirely included under the radii of the different buffers. When the buffer radius crossed a polygon, a coefficient was applied to the initial population size of the complete polygon, a coefficient that was estimated based on the area of the polygon covered by the buffer radius, as explained in Section 2. This approach assumes that the distribution of the population is homogeneous across a polygon, which could lead to small errors if this is not the case. However, this small error is certainly limited for two reasons. First, the most populated urban areas also correspond to the urban sections that tend to have a more homogeneous distribution of the population. Moreover, the MGN urban polygons have small areas, and therefore, as the buffer radius increases, more MGN polygons will be completely included within the buffers—reducing the need to apply the correction. For example, within a radius of 500 m around the Eternit plant in Barranquilla in 2005, 0 MGN polygons of urban sections out of 7 are entirely within the buffer (i.e., the correction had to be applied to seven polygons—100%), 1 out of 12 are entirely within the 1000 m radius buffer, 21 out of 49 are entirely within the 2000 m radius buffer, 209 out of 259 are entirely within the 5000 m radius buffer, and 711 out of 750 are entirely within the 10,000 m radius buffer (i.e., the correction was applied to 39 polygons—5.2%). Second, rural sections or sectors—although more extensive—are also much less populated than the urban sections or sectors; the error will, therefore, be limited in this case as well.

Another limitation is a possible underestimation of the population at risk as a result of certain sectors without demographic data (Supplementary Material, Figure S3a,b). Some scenarios—especially in 2005—have as much as 50% of the polygons without data in rural areas (i.e., the mine in Campamento with one polygon out of two). For the same year, in urban areas, the largest percentage of the polygons with no data was 21.4%, in the 1000 m band around Eternit, Yumbo. Several official communications with the DANE have helped explain this phenomenon, which was the result of personal safety concerns of census officials in these areas that prevented them from using, in some cases, geolocation equipment to record census information. In addition, it was identified that some urban sections—in urban areas, as confirmed by satellite images—sometimes appeared to not have resident populations. The DANE indicates, from an example we provided, that some areas correspond to areas with buildings that are not housing units (e.g., industrial zones), which could explain why sometimes these areas do not have residents. This is notably the case near Eternit, Yumbo. Nevertheless, this lack of data tended to decrease between the censuses, decreasing from 4.6% of polygons without data in the 10,000 m band for all the sites studied in 2005 to 1.4% in 2018 (Supplementary Material, Figure S3a,b). Finally, it is possible to note a relative drop in the population in the 500, 1000, and 10,000 m radii around Eternit, Yumbo between 2005 and 2018, which the DANE explains resulted from the degree of geographical precision.

Another aspect to mention is the narrow temporal window of the analysis conducted (years 2005 to 2018). Indeed, since the asbestos industry began operations in Colombia in 1942, it would have been interesting to replicate this work with previous censuses. The DANE confirmed that only these two censuses are available online. Furthermore, for previous censuses, the microdata from the 1993 General Population Census is not easily available and can only be consulted in the specialized external processing room of the DANE. Thus, in future studies, it would be important to determine the feasibility of including the 1993 census in the analysis.

Another limitation of this method was the use of concentric areas in the analysis. Although it was based on distances reported in studies conducted in other regions of the world, the perfectly concentric character of the potential dispersion of asbestos fibers is geometrically convenient. The topographical context and local meteorology play a role in the dispersion of asbestos fibers, and as a result, in the risk of asbestos exposure. The importance of prevailing winds in the simulation of asbestos dispersion from factories producing asbestos cement products [21,46] or asbestos textiles [41] has been clearly established and indicates that such dispersion is not necessarily concentric.

Finally, this work—recognizing the significance that the potential dispersion of asbestos from industrial sources may play in its exposure to the population—must not obscure other exposure scenarios. As has been reported by other authors in the past, a recent study reaffirms the possibility of having other sources of asbestos exposure in the area surrounding an asbestos facility, including both domestic and occupational settings [29].

In the case of Sibaté (Colombia), where an asbestos facility operated for more than seven decades, massive disposal of waste contaminated with friable asbestos in the urban area of the municipality was identified [39,47,48], which is something that may have occurred around other asbestos processing facilities. This point is particularly critical in a country like Colombia, where waste regulations are fairly recent [49,50,51] and the degree of compliance and enforcement by the authorities is unknown. In this context, it is important to implement health surveillance programs for asbestos workers and their families, determine if there are still active sources that could be releasing asbestos fibers into the environment, and study the potential presence of contaminated sites because of the improper disposal of residues contaminated with asbestos, especially for the areas where the asbestos facilities and the mine operated in Colombia. Because of the lack of enforcement and surveillance of asbestos processing facilities in low- and middle-income countries, the vast majority of studies have been conducted in high–income countries.

## 5. Conclusions

In this study, we estimated the number of people living in Colombia in close proximity to asbestos processing facilities and the only asbestos mine in the country (up to a distance of 10 km). Within these distances, studies conducted in other parts of the world have found an increased risk of ARD, especially mesothelioma. However, these risk estimates cannot be directly applied to the Colombian population because local aspects, such as meteorological conditions, particular attributes of each plant (i.e., type of products manufactured, volumes and asbestos types used, production process, and emission control strategies in place), and local topography, among others, can modify the risk experienced by the population living around each plant. The results of the current study reaffirm the importance of implementing mesothelioma registries and other surveillance strategies for asbestos-related diseases in Colombia, especially in the regions where asbestos processing plants or the mine are located, and the urgent need to develop and implement a National Plan for the Elimination of Asbestos-related Diseases.

## Figures and Tables

**Table 1:** The six facilities and the mine included in this study.

Name	Municipality (and Department)	Type of Facility	Production	Starting Date
Eternit Colombiana SA	Sibaté (Cundinamarca)	Plant	Asbestos cement	1942
Eternit Atlántico SA	Barranquilla (Atlántico)	Plant	Asbestos cement	1944
Eternit Pacífico SA	Yumbo (Valle del Cauca)	Plant	Asbestos cement	1944
Incolbest SA	Bogotá (Bogotá DC)	Plant	Friction products	1960
Etex Colombia SA	Manizales (Caldas)	Plant	Asbestos cement	1967
Toptec SA	Manizales (Caldas)	Plant	Asbestos cement	1982
Minera Las Brisas	Campamento (Antioquia)	Mine	Chrysotile	1980

**Table 2:** Summary of distances used in the current study.

Article Reference	Asbestos-Related Diseases Studied	Sources of Environmental Exposure	Asbestos Type	Risk-Related Indicator	Relationship between Risk and Distance to the Plant						Observations
					<500 m	500 m	1000 m	2000 m	5000 m	10,000 m	
Vimercati et al., 2020	Epithelioid mesothelioma	Asbestos cement plant	Amosite and crocidolite: 20%; chrysotile: 80%	(1) Adjusted risks of EM (OR) by distance and exposure length (2) Adjusted risks of EM (OR) by distance and latency length		(<550 m) (1) OR = 1.47 (0.262–8.248) // (2) OR = 1.771 (0.32–9.805). *Compared to people living at >1000 m*	(550–1000 m) (1) OR = 1.87 (0.353–9.905) // (2) OR = 2.038 (0.371–11.19). *Compared to people living at >1000 m*				Apulia Mesothelioma Registry. There is an exposure reconstruction. They looked at the records of 2236 cases between 1989 and 2019. The 71 cases analyzed were only environmentally exposed. The distance of 550–1000 m is a finding of the study, based on the living locations of the 71 cases. 3000 m was the largest distance that cases lived from the factory, but there was no risk estimate for 3000 m.
Tarrés et al., 2012	Malignant pleural mesothelioma	Asbestos cement plant	Amosite: 5%; crocidolite: 15%; chrysotile: 80%	Incidence rate ratio	(<500 m) IRR = 12.92 (10.77–15.33)			(>500–2000 m) IRR = 0.70 (0.28–1.44)		(>2000–10,000 m) IRR = 0.23 (0.02–0.72)	Clinical and epidemiological data were recorded for the 24 pleural mesothelioma cases. Places of residence were obtained from primary healthcare documentation. Concentric circles were used for the analysis.
Kurumatani, Kumagai, 2008	Pleural and peritoneal mesotheliomas + pleural cancers (on death certificate)	Asbestos cement plant	Crocidolite and chrysotile	SMR	SMR Men (<300 m) = 13.9 (5.6–28.7) // SMR Women (<300 m) = 41.4 (15.2–90.1)	SMR Men (300 to 600 m) = 5.6 (2.9–9.8) // SMR Women (300 m to 600 m) = 11.3 (4.5–23.3)	SMR Men (600 to 900 m) = 2.1 (0.8–4.3) // SMR Women (600 m to 900 m) = 7.2 (2.9–14.8)				90 cases. Distances were analyzed with 300 m increments.
Musti et al., 2009	Malignant mesothelioma	Asbestos cement plant	Amosite: 5%; crocidolite: 15%; chrysotile: 80%	OR adjusted by sex and age	(0–500 m) OR = 5.29 (1.18–23.74). *Compared to a distance of >2000 m*		(500–1000 m) OR = 1.49 (0.58–3.82) // (1000–1500 m) OR = 1.27 (0.49–3.71). *Compared to a distance of >2000 m*	(1500–2000 m) OR = 2.31 (0.88–6.06). *Compared to a distance of >2000 m*			Case–control study. Regional Mesothelioma Register–Bari. 48 cases of malignant mesothelioma, non-occupationally exposed, and 273 controls.
Maule et al., 2007	Malignant pleural mesothelioma	Asbestos cement plant	Crocidolite: 10%; “airborne emissions from the AC plant included both chrysotile and crocidolite fibers”	OR adjusted by sex, age, domestic exposure, and relatives that work in the AC plant					(0–3000 m ) OR = 6.8 (2.4–19.5) // (3000–5000 m) OR = 12.5 (3.7–42.2)	(5000–7000 m ) OR = 3.9 (1.2–12.6) // (7000–9000 m) OR = 4.2 (1.1–16.5)	Case–control study. 103 cases, 272 controls. Casale Monferrato. Piedmont Mesothelioma Registry.
Airoldi et al., 2021	Malignant pleural mesothelioma	Asbestos cement plant	Crocidolite: 10%	OR adjusted by age, sex, type of interview, and domestic exposure					(0–5000 m) OR = 12.66 (4.19–38.28). *Compared to a distance of >15,000 m*	(5000–10,000 m) OR = 7.15 (1.87–27.36). *Compared to a distance of >15,000 m*	Case–control study. 200 cases, 348 controls. Casale Monferrato.
Magnani et al., 2000	Malignant pleural mesothelioma	Asbestos cement plants; asbestos textiles, shipyards, or brakes factories	Unspecified	OR adjusted by center, sex, and age				“A high risk was observed for high probability of environmental exposure (11.5 (3.47–38.2))—that is, subjects who had lived for some time within 2000 m of a mine or asbestos facility”			Histologically confirmed cases. Exposure classification of cases. High risk of exposure (not disease).

**Table 3:** Number of people living around the seven main asbestos processing/mining sites in Colombia—2005.

Age Groups (Years)	Incolbest—Bogotá					Eternit—Sibaté					Eternit—Yumbo					Eternit—Barranquilla					Las Brisas—Campamento					Toptec—Manizales					Colombit (Etex)—Manizales				
	500 m	1000 m	2000 m	5000 m	10,000 m	500 m	1000 m	2000 m	5000 m	10,000 m	500 m	1000 m	2000 m	5000 m	10,000 m	500 m	1000 m	2000 m	5000 m	10,000 m	500 m	1000 m	2000 m	5000 m	10,000 m	500 m	1000 m	2000 m	5000 m	10,000 m	500 m	1000 m	2000 m	5000 m	10,000 m
0–4	354	1872	10,012	58,109	283,057	4	15	201	17,846	49,988	47	547	2203	5719	28,547	65	864	3339	33,099	134,411	0	0	0	35	303	308	911	1210	5733	24,391	20	53	93	1541	19,273
5–9	377	1963	11,008	61,771	312,962	4	16	251	20,294	57,551	55	667	2574	6580	32,572	68	873	3412	34,829	133,712	0	0	0	41	364	361	1108	1493	7058	28,602	28	73	116	1891	22,871
10–14	347	1779	10,207	56,974	300,387	4	17	229	19,992	56,800	66	667	2694	6832	33,313	79	992	3890	35,847	130,874	0	0	0	37	341	377	1134	1531	7944	32,080	28	71	119	1932	25,547
15–19	274	1580	9329	50,759	281,226	4	16	185	17,523	47,645	50	549	2303	6221	31,235	89	1071	4610	38,786	132,424	0	0	0	29	281	411	1278	1717	9194	33,149	25	69	112	2092	26,292
20–24	311	1929	10,605	55,085	316,058	6	20	196	18,379	46,706	50	567	2094	5840	31,754	89	1063	5294	43,047	137,290	0	0	0	18	187	468	1422	1918	10,407	33,886	19	53	91	2238	26,877
25–29	345	2066	10,354	55,095	307,855	3	10	149	14,911	40,169	41	493	1753	5267	29,665	171	1145	4376	36,814	119,697	0	0	0	15	146	470	1400	1799	7830	28,011	22	56	91	2117	21,967
30–34	395	1948	9573	51,653	274,411	2	8	153	12,857	36,185	37	453	1688	4891	26,811	67	812	3468	30,844	100,196	0	0	0	16	145	382	1149	1487	6659	24,052	23	57	86	1797	19,249
35–39	352	1725	9076	49,904	268,013	3	10	149	13,557	38,813	43	407	1720	4907	26,769	79	903	3760	31,586	100,699	0	0	0	13	154	377	1092	1459	7416	26,567	27	70	104	1834	21,088
40–44	279	1422	7855	45,254	253,778	2	9	130	12,994	36,783	46	410	1629	4653	25,562	82	956	3902	32,707	97,947	0	0	0	15	160	332	1032	1383	7478	26,752	22	62	98	1722	20,978
45–49	173	1013	6136	35,323	207,639	2	7	87	11,957	30,749	34	329	1247	3611	20,955	70	755	3458	28,280	80,426	0	0	0	12	140	349	1055	1413	6815	24,682	14	38	68	1668	18,980
50–54	131	831	4414	25,834	160,060	2	6	69	9077	22,995	21	251	928	2832	16,817	56	644	2823	23,416	63,545	0	0	0	12	115	345	1068	1392	5965	20,724	11	28	54	1601	15,981
55–59	83	673	3373	18,758	122,064	1	5	60	5768	15,313	15	190	669	2171	12,823	37	452	2124	18,723	48,676	0	0	0	8	87	280	851	1114	4716	15,988	8	23	42	1297	12,328
60–64	66	505	2428	13,081	89,080	1	3	48	3490	10,056	18	162	501	1581	9112	28	365	1555	13,875	34,896	0	0	0	9	83	173	554	718	3482	11,904	10	25	38	878	9256
65–69	45	305	1783	8923	63,725	1	4	51	2571	7701	9	118	460	1415	7429	22	292	1285	11,980	29,625	0	0	0	6	72	130	404	539	2773	9759	7	18	29	664	7664
70–74	35	202	1115	6061	46,247	0	2	34	1617	5389	7	84	321	1017	5363	22	243	1067	9286	22,328	0	0	0	3	64	89	281	371	2162	7652	5	13	21	452	5904
75–79	25	134	793	4305	32,006	0	2	16	1022	3171	5	47	191	714	3662	18	218	915	7769	17,031	0	0	0	3	37	59	203	268	1594	5643	2	4	9	313	4339
80+	15	118	704	3912	28,352	0	1	16	907	2640	5	43	184	689	3480	17	225	989	8546	17,953	0	0	0	3	45	52	169	232	1455	5067	2	5	9	276	3887
TOTAL	3606	20,065	108,768	600,803	3,346,920	40	150	2025	184,763	508,656	547	5985	23,161	64,938	345,868	1059	11,875	50,267	439,434	1,401,729	0	0	2	274	2724	4963	15,110	20,044	98,680	358,909	273	718	1178	24,312	282,479

**Table 4:** Number of people living around the seven main asbestos processing/mining sites in Colombia—2018.

Age Groups (Years)	Incolbest—Bogotá					Eternit—Sibaté					Eternit—Yumbo					Eternit—Barranquilla					Las Brisas—Campamento					Toptec—Manizales					Colombit (Etex)—Manizales				
	500 m	1000 m	2000 m	5000 m	10,000 m	500 m	1000 m	2000 m	5000 m	10,000 m	500 m	1000 m	2000 m	5000 m	10,000 m	500 m	1000 m	2000 m	5000 m	10,000 m	500 m	1000 m	2000 m	5000 m	10,000 m	500 m	1000 m	2000 m	5000 m	10,000 m	500 m	1000 m	2000 m	5000 m	10,000 m
0–4	364	1696	7518	43,828	217,519	1	3	163	20,621	68,139	30	359	1718	4717	16,713	62	556	2097	22,997	102,243	4	19	43	142	557	255	735	918	4949	19,201	10	26	32	1082	15,300
5–9	384	1781	8071	47,133	236,397	1	4	165	22,373	74,775	32	384	1839	5107	18,411	66	611	2181	23,962	106,250	7	30	69	215	800	278	818	1012	5377	21,197	14	38	48	1182	16,900
10–14	422	1832	8657	50,446	251,234	1	3	179	23,495	77,893	38	455	2163	5917	20,901	60	602	2210	25,173	108,512	8	35	84	269	949	318	948	1203	6092	24,504	20	48	59	1393	19,581
15–19	500	2124	9908	57,992	290,521	1	3	185	25,678	84,035	49	540	2390	6610	23,432	74	736	2806	29,569	121,016	7	32	75	251	940	380	1122	1425	7732	29,767	19	51	66	1670	23,560
20–24	612	2751	12,639	72,384	363,729	1	3	204	27,338	91,143	49	558	2533	7058	26,743	88	812	3166	32,150	124,179	7	31	64	176	699	442	1335	1716	9470	34,697	19	53	66	2004	27,635
25–29	602	2775	12,394	71,283	354,252	1	4	178	24,513	87,213	48	511	2396	6774	26,444	79	755	2950	30,751	119,170	7	30	60	153	601	382	1147	1464	8402	32,040	18	44	57	1710	25,327
30–34	537	2366	10,508	64,642	315,266	1	4	198	23,219	81,174	41	454	2073	5985	24,740	66	638	2561	28,352	108,759	3	15	34	126	554	378	1167	1469	7695	28,903	15	42	52	1694	22,690
35–39	498	2225	9583	61,954	299,604	1	4	181	22,280	74,720	34	444	2018	5920	24,664	60	666	2608	28,398	105,724	4	16	37	126	572	479	1355	1695	8207	29,191	20	47	57	1950	22,910
40–44	434	1846	8051	52,008	252,717	0	2	143	17,840	59,484	29	385	1719	5111	21,614	67	626	2313	24,079	87,861	4	18	40	123	453	388	1119	1419	7135	24,764	20	49	61	1667	19,698
45–49	418	1814	7912	48,521	239,694	1	2	125	16,610	55,145	29	357	1706	5097	21,363	60	605	2190	23,427	82,756	3	15	34	108	474	413	1135	1422	7447	26,039	20	48	58	1666	20,657
50–54	387	1643	7618	45,803	235,627	1	2	123	15,816	51,432	35	355	1686	5014	21,691	60	618	2480	25,432	83,722	3	14	32	96	430	387	1103	1401	7926	27,675	16	44	53	1650	21,779
55–59	271	1233	6129	37,785	202,030	1	3	100	13,587	43,038	34	322	1346	4131	19,152	58	583	2363	24,362	75,116	3	12	26	85	410	360	1065	1334	7529	26,288	10	29	37	1532	20,345
60–64	193	917	4589	27,429	151,308	0	2	67	10,926	32,412	24	248	1037	3311	15,599	47	480	1997	20,502	59,893	2	8	19	68	309	353	1027	1312	6612	22,510	8	21	27	1469	17,386
65–69	113	659	3187	18,836	109,189	0	1	47	7217	21,564	13	173	748	2416	11,707	28	332	1460	16,169	44,985	2	9	19	52	238	292	871	1095	5224	17,414	6	14	18	1218	13,484
70–74	78	515	2283	12,310	75,028	0	2	44	4263	13,396	11	130	531	1745	8710	24	268	1094	11,618	31,007	1	4	10	37	185	205	619	791	3812	12,660	7	17	19	897	9899
75–79	57	351	1532	8187	52,393	0	1	37	2605	8508	12	106	417	1257	6114	17	214	843	8850	22,602	1	4	7	22	143	133	407	514	2637	8796	5	13	17	594	6920
80–84	30	189	895	5070	34,419	0	0	19	1455	4963	4	60	237	830	4116	16	162	609	6366	15,388	0	1	4	16	95	80	228	288	1784	5935	4	10	12	341	4611
85–89	21	88	453	2575	18,572	0	0	9	755	2521	3	32	121	452	2152	10	96	376	3752	8729	0	1	1	6	44	38	116	155	1000	3312	2	5	6	179	2566
90–94	4	27	163	1035	7173	0	0	3	273	888	1	9	59	223	880	4	30	148	1733	3980	0	0	0	1	15	16	52	68	450	1378	0	1	1	74	1071
95–99	2	14	55	279	1893	0	0	1	68	243	0	4	16	54	226	1	14	56	559	1288	0	0	0	0	2	2	7	8	89	301	0	0	0	11	233
100+	1	4	15	118	762	0	0	0	47	150	0	0	4	17	66	0	5	20	243	536	0	0	0	0	1	1	3	3	29	88	0	0	0	4	65
TOTAL	5927	26,851	122,161	729,618	3,709,324	12	45	2172	280,981	932,838	516	5886	26,758	77,746	315,439	948	9409	36,527	388,444	1,413,714	66	292	658	2071	8472	5582	16,378	20,715	109,599	396,661	234	599	746	23,986	312,615

**Table 5:** Populations living in the vicinity of two plants in 2005 (Etex and Toptec—Manizales; Incolbest—Bogotá and Eternit—Sibaté).

Age Groups (Years)	ETEX and TOPTEC					INCOLBEST and ETERNIT SIBATE				
	500 m	1000 m	2000 m	5000 m	10,000 m	500 m	1000 m	2000 m	5000 m	10,000 m
0–4	N/A	N/A	7	1456	19,240	N/A	N/A	N/A	N/A	3799
5–9	N/A	N/A	6	1800	22,840	N/A	N/A	N/A	N/A	4324
10–14	N/A	N/A	9	1839	25,513	N/A	N/A	N/A	N/A	4026
15–19	N/A	N/A	7	2006	26,263	N/A	N/A	N/A	N/A	3557
20–24	N/A	N/A	8	2170	26,854	N/A	N/A	N/A	N/A	3697
25–29	N/A	N/A	7	2050	21,941	N/A	N/A	N/A	N/A	3436
30–34	N/A	N/A	5	1740	19,230	N/A	N/A	N/A	N/A	3146
35–39	N/A	N/A	5	1768	21,062	N/A	N/A	N/A	N/A	3000
40–44	N/A	N/A	7	1661	20,957	N/A	N/A	N/A	N/A	2576
45–49	N/A	N/A	7	1614	18,961	N/A	N/A	N/A	N/A	1896
50–54	N/A	N/A	6	1552	15,965	N/A	N/A	N/A	N/A	1578
55–59	N/A	N/A	3	1261	12,318	N/A	N/A	N/A	N/A	1266
60–64	N/A	N/A	2	849	9249	N/A	N/A	N/A	N/A	916
65–69	N/A	N/A	2	640	7659	N/A	N/A	N/A	N/A	629
70–74	N/A	N/A	1	437	5901	N/A	N/A	N/A	N/A	408
75–79	N/A	N/A	1	303	4337	N/A	N/A	N/A	N/A	253
80+	N/A	N/A	1	268	3885	N/A	N/A	N/A	N/A	223
TOTAL	N/A	N/A	82	23,416	282,175	N/A	N/A	N/A	N/A	38,728

**Table 6:** Populations living in the vicinity of two plants in 2018 (Etex and Toptec—Manizales; Incolbest—Bogotá and Eternit—Sibaté).

Age Groups (Years)	ETEX and TOPTEC					INCOLBEST and ETERNIT SIBATE				
	500 m	1000 m	2000 m	5000 m	10,000 m	500 m	1000 m	2000 m	5000 m	10,000 m
0–4	N/A	N/A	2	1077	15,297	N/A	N/A	N/A	N/A	3661
5–9	N/A	N/A	3	1176	16,895	N/A	N/A	N/A	N/A	4070
10–14	N/A	N/A	5	1390	19,575	N/A	N/A	N/A	N/A	4170
15–19	N/A	N/A	6	1664	23,555	N/A	N/A	N/A	N/A	4624
20–24	N/A	N/A	4	1999	27,627	N/A	N/A	N/A	N/A	5256
25–29	N/A	N/A	4	1700	25,323	N/A	N/A	N/A	N/A	5144
30–34	N/A	N/A	3	1690	22,687	N/A	N/A	N/A	N/A	4411
35–39	N/A	N/A	4	1944	22,908	N/A	N/A	N/A	N/A	3926
40–44	N/A	N/A	5	1663	19,694	N/A	N/A	N/A	N/A	3337
45–49	N/A	N/A	3	1662	20,651	N/A	N/A	N/A	N/A	3142
50–54	N/A	N/A	2	1647	21,775	N/A	N/A	N/A	N/A	2840
55–59	N/A	N/A	3	1529	20,339	N/A	N/A	N/A	N/A	2260
60–64	N/A	N/A	2	1465	17,384	N/A	N/A	N/A	N/A	1650
65–69	N/A	N/A	2	1217	13,483	N/A	N/A	N/A	N/A	1258
70–74	N/A	N/A	1	896	9898	N/A	N/A	N/A	N/A	922
75–79	N/A	N/A	1	592	6920	N/A	N/A	N/A	N/A	578
80–84	N/A	N/A	1	339	4608	N/A	N/A	N/A	N/A	323
85–89	N/A	N/A	0	179	2565	N/A	N/A	N/A	N/A	180
90–94	N/A	N/A	0	74	1071	N/A	N/A	N/A	N/A	59
95–99	N/A	N/A	0	11	233	N/A	N/A	N/A	N/A	17
100+	N/A	N/A	0	4	65	N/A	N/A	N/A	N/A	2
TOTAL	N/A	N/A	51	23,916	312,554	N/A	N/A	N/A	N/A	51,831

**Table 7:** Number of people living in the vicinity of the 7 sites analyzed.

Year	500 m	1000 m	2000 m	5000 m	10,000 m
2005	10,489	53,904	205,363	1,389,788	5,926,382
2018	13,285	59,459	209,685	1,588,528	6,724,677

**Table 8:** Number of cases of pleural, peritoneal, and pericardial mesotheliomas attended by health providers from 2015 to the first half of 2021 (as of 30 June 2021).

Municipality	C450—Pleural Mesothelioma		C451—Mesothelioma of Peritoneum		C452—Mesothelioma of Pericardium	
	Females	Males	Females	Males	Females	Males
Barranquilla	38	25	52	15	45	19
Bogotá	97	163	56	11	4	5
Campamento	-	-	-	-	-	-
Manizales	3	6	6	1	-	1
Sibaté	5	16	-	-	-	-
Yumbo	-	3	-	1	-	-
Total	143	213	114	28	49	25

Source: SISPRO—accessed on 5 October 2021.

## Data Availability

Data bases used can be found at: 1—DANE. Censos Nacionales de Población y Vivienda. 2020. Available online: http://systema59.dane.gov.co/bincol/rpwebengine.exe/PortalAction?lang=esp (accessed on 3 November 2021). 2—DANE. Marco Geoestadistico Nacional—MGN. 2020. Available online: https://geoportal.dane.gov.co/servicios/descarga-y-metadatos/descarga-mgn-marco-geoestadistico-nacional/ (accessed on 3 November 2021).

## Supplementary Materials

The following are available online at https://www.mdpi.com/article/10.3390/ijerph182413297/s1, Figure S1: Protocol to access and process census data from Colombia, Figure S2: Overview of the GIS procedure, and Figure S3a: Number of polygons without data in 2005 for each distance scenario, and Figure S3b: Number of polygons without data in 2018 for each distance scenario.
