# Supplementary material for: Using GIS to Estimate Population at Risk Because of Residence Proximity to Asbestos Processing Facilities in Colombia
Source: Int J Environ Res Public Health. 2021 Dec 17;18(24):13297. doi: 10.3390/ijerph182413297 (PMC8703708; doi:10.3390/ijerph182413297)
Supplement: Supplementary file 1 [file ijerph-18-13297-s001.zip › Supplementary material 3.pdf]

Supplementary material 3

Figure S3-a: Number of polygons without data in 2005 for each distance scenario

	number of polygons without demographic data / total number of polygons - 500m	number of polygons without demographic data / total number of polygons - 1,000m	number of polygons without demographic data / total number of polygons - 2,000m	number of polygons without demographic data / total number of polygons - 5,000m	number of polygons without demographic data / total number of polygons - 10,000m
Incolbest - Bogotá	0 / 11	1 / 21 (4.8 %)	1 / 53 (1.9%)	7 / 226 (3.1%)	17 / 1,210 (1.4%)
Eternit - Sibaté	0 / 1	0 / 2	0 / 7	18 / 72 (25%)	34 / 298 (11.4%)
Las Brisas - Campamento	1 / 2 (50%)	1 / 2 (50%)	1 / 2 (50%)	2 / 6 (30%)	5 / 15 (30%)
Eternit - Yumbo	0 / 5	3 / 14 (21.4%)	5 / 32 (15.6%)	7 / 67 (10.4%)	31 / 209 (14.8%)
Eternit - Barranquilla	0 / 7	1 / 13 (7.7%)	2 / 51 (3.9%)	4 / 264 (1.5%)	29 / 758 (3.8%)
Toptec - Manizales	0 / 9	0 / 14	0 / 17	2 / 92 (2.2%)	14 / 260 (5.4%)
Etex - Manizales	0 / 5	0 / 5	0 / 6	2 / 26 (7.7%)	6 / 211 (2.9%)
All plants	1 / 40 (2,5%)	5 / 71 (7%)	9 / 168 (5.4%)	42 / 753 (5.6%)	136 / 2,961 (4.6%)

Figure S3-b: Number of polygons without data in 2018 for each distance scenario

	number of polygons without demographic data / total number of polygons - 500m	number of polygons without demographic data / total number of polygons - 1,000m	number of polygons without demographic data / total number of polygons - 2,000m	number of polygons without demographic data / total number of polygons - 5,000m	number of polygons without demographic data / total number of polygons - 10,000m
Incolbest - Bogotá	0 / 11	0 / 21	0 / 53	5 / 227 (2.2%)	12 / 1,217 (1%)
Eternit - Sibaté	0 / 1	0 / 3	0 / 9	0 / 78	2 / 312 (0.6%)
Las Brisas - Campamento	0 / 2	0 / 3	0 / 4	0 / 11	0 / 30
Eternit - Yumbo	0 / 5	1 / 14 (7.1%)	4 / 32 (12.5%)	7 / 73 (1%)	11 / 227 (4.8%)
Etemit - Barranquilla	0 / 7	0 / 13	2 / 50 (4%)	2 / 278 (0.7%)	10 / 783 (1.3%)
Toptec - Manizales	0 / 9	0 / 15	0 / 19	0 / 97	4 / 280 (1.4%)
Etex - Manizales	0 / 5	0 / 6	0 / 7	0 / 29	3 / 228 (1.3%)
All plants	0 / 40 (0%)	1 / 75 (1.3%)	6 / 174 (3.4%)	14 / 793 (1.8%)	42 / 3,077 (1.4%)
