# Supplementary figures and images for: Using GIS to Estimate Population at Risk Because of Residence Proximity to Asbestos Processing Facilities in Colombia
Source: Int J Environ Res Public Health. 2021 Dec 17;18(24):13297. doi: 10.3390/ijerph182413297 (PMC8703708; doi:10.3390/ijerph182413297)

## Supplementary material 1

Figure S1: Protocol to access and process census data from Colombia

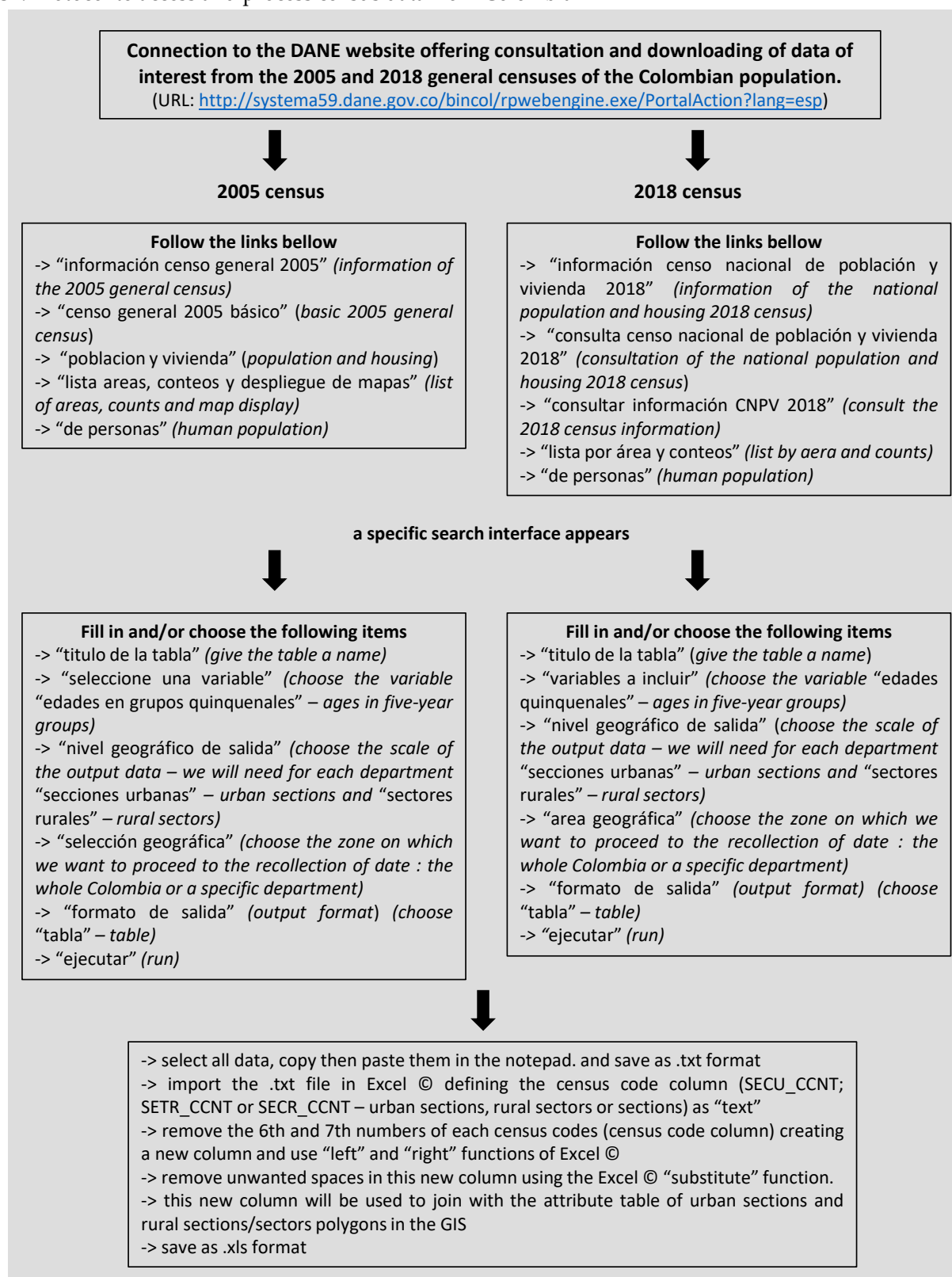

Supplement: Supplementary file 1 [file ijerph-18-13297-s001.zip › Supplementary material 1.pdf]

## Supplementary material 2

Figure S2: Overview of the GIS procedure

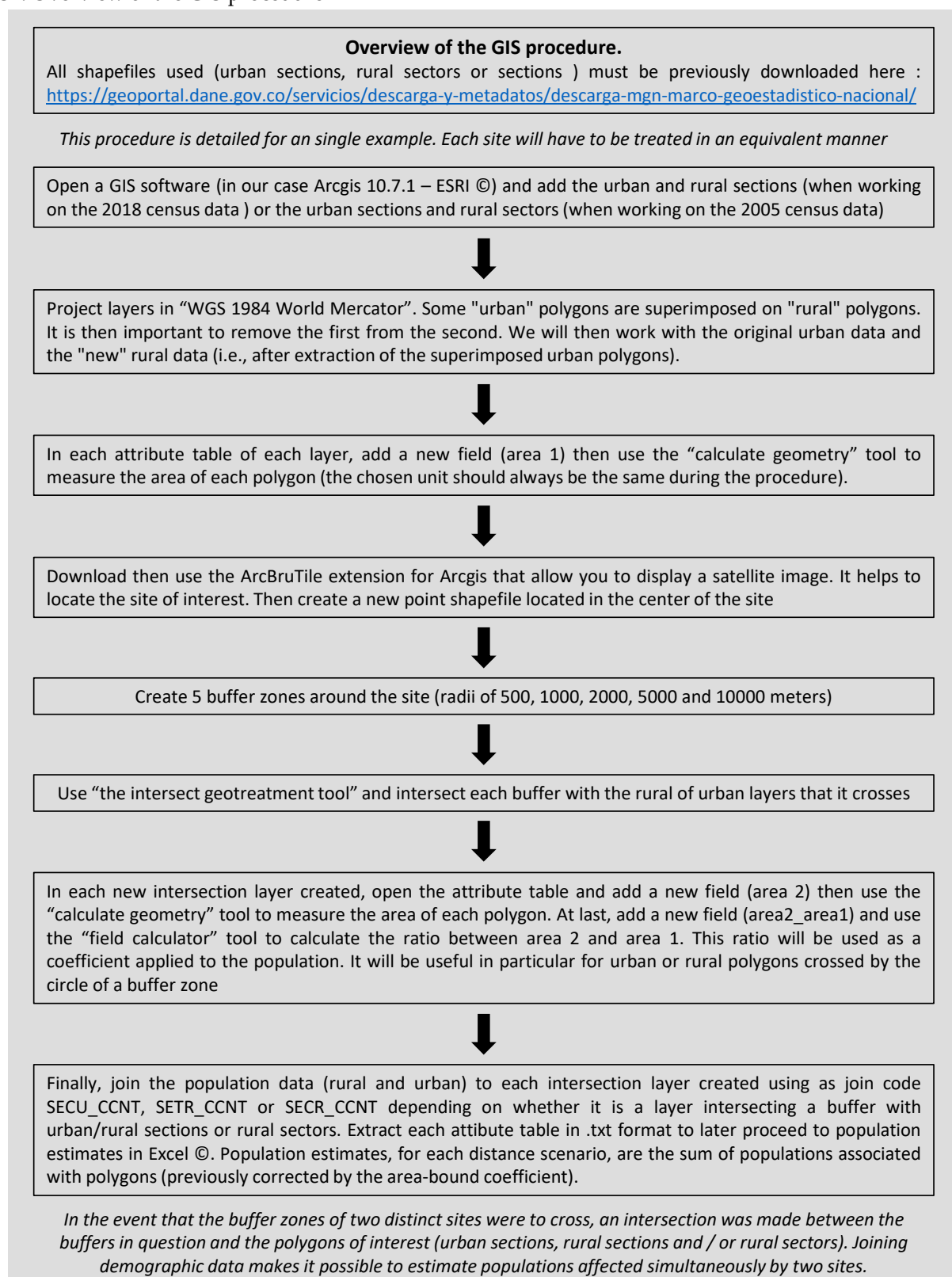

Supplement: Supplementary file 1 [file ijerph-18-13297-s001.zip › Supplementary material 2.pdf]
